# Incidence of diabetic foot ulcer and its predictors among adult diabetes patients in Northern Ethiopia: a retrospective cohort study

**DOI:** 10.1038/s41598-025-19253-7

**Published:** 2025-10-10

**Authors:** Yohannes Terefe Ayalew, Behailu Tariku Derseh, Enguday Demeke Gebeyaw, Hailemelekot Bekele Kebede

**Affiliations:** School of Public Health, Asrat Woldeyes Health Science Campus, Debre Berhan University, Debre Berhan, Ethiopia

**Keywords:** Diabetic foot ulcer, Incidence, Retrospective cohort, Diabetes complications, Epidemiology, Epidemiology, Lifestyle modification

## Abstract

Diabetic foot ulcer (DFU) is a leading cause of lower limb amputation worldwide. The rate of diabetes-related amputations is rising in Ethiopia. However, the incidence of diabetic foot ulcer and its predictors were less studied in Ethiopia, particularly in the study area. An institution-based retrospective cohort study was conducted. Systematic random sampling was used to select a sample of 321 patients out of 1050 diabetes patients. Data were entered into EpiData and analysed using SPSS. Kaplan-Meier survivor functions were used for survival analysis. Cox proportional hazard regression was used to identify predictors, and multivariable analysis was declared with a *p* ≤ 0.25. The incidence of diabetic foot ulcers was 1.01 per 100 person-years (95%: CI, 0.74–1.40. Obesity (AHR = 2.94: 95% CI: 1.11–7.79), neuropathy (AHR = 2.94: 95% CI: 1.47–5.90), rural residence (AHR = 2.73: 95% CI: 1.27–5.88), hypertension (AHR = 5.61: 95% CI: 2.49–12.90), age ≥ 70 years (AHR = 15.03: 95% CI: 3.58–63.10) and abnormal high-density lipoprotein (AHR = 3.71: 95% CI: 1.81–7.62) increased the hazard of diabetic foot ulcer. The overall incidence of diabetic foot ulcers was relatively low in this cohort. We recommend targeted intervention, including regular screening, patient education, and early therapeutic management.

**Supplementary Information:**

The online version contains supplementary material available at 10.1038/s41598-025-19253-7.

## Introduction

Diabetes mellitus (DM) is a metabolic disorder characterised by the body’s inability to produce or properly use insulin. It is characterised by high blood sugar (hyperglycemia) and also gradually causes various chronic complications that affect nearly every organ. DM is typically classified into Type 1 diabetes, Type 2 diabetes, Specific types of diabetes due to other causes (e.g., monogenic diabetes syndromes, etc.), and Gestational diabetes mellitus (GDM)^1^.

A diabetic foot ulcer (DFU) is a non-traumatic skin lesion of the foot in a person with DM^2^. DFU deteriorates the quality of life and is the primary cause of leg amputations. Mortality ranges from 24.6% at 5 years and 45.4% at 10 years^3–7^. Recent studies continue to highlight the burden of DFU-related mortality. In Indonesia, it was reported that severe infection and coronary artery disease increased the mortality risk in DFU patients by five times^8^. Another cohort found that high-risk foot status, age ≥ 60, and HbA1c ≥ 7% significantly increased the risk of amputation or death within three years^9^. During the COVID-19 pandemic, DFU patients experienced more severe infections, delayed care, and a rise in major amputations, indicating poorer outcomes and increased mortality risk^10^. Furthermore, nearly a quarter of all diabetes healthcare expenditure is due to foot complications^11^.

Several factors contribute to the development of diabetic foot ulcers (DFUs), including sociodemographic, clinical, biological, and behavioural characteristics. These may involve aspects such as patient demographics, comorbidities, lipid profile, and lifestyle practices, including foot care, physical activity, and substance use^12–15^.

The global prevalence of DFU accounts for 6.3%, with a higher in males and type 2 DM, while the prevalence in the African region is slightly higher, at 7.2%^16^, and foot complications like ulceration, infection, or gangrene cause long-term disability and premature death for up to 25% of patients during their lifetime^17–19^. In Ethiopia, a systematic review and meta-analysis indicated that 12.98% of diabetes patients develop the onset of DFU^6^.

In Ethiopia, where diabetes prevalence is increasing, DFU is a significant but under-researched complication, particularly in rural areas. Most Ethiopian studies have used cross-sectional designs^13,15,20^, limiting temporal assessment of risk factors. Furthermore, limited data exist on the incidence and predictors of DFU using long-term follow-up methods, and no such study has been conducted in the present study setting. Our retrospective cohort study fills these gaps by assessing the incidence of diabetic foot ulcer and its predictors in adult diabetes patients in Debre Berhan Comprehensive Specialised Hospital, North Shewa, Amhara, Ethiopia, to generate evidence for early detection and targeted prevention.

## Materials and methods

### Study design, setting, and period

An institutional-based retrospective cohort study was conducted from January 1, 2005 and ended on December 31, 2021. The study was conducted in Debre Berhan Comprehensive Specialised Hospital in Debre Berhan town. Debre Berhan is the capital city of North Shewa, one of the 13 zones of the Amhara regional state, which is located 130 Km north of Addis Ababa, Ethiopia. The town has 1 recognised comprehensive specialised Hospital. Currently, the hospital has 496 health professionals. The monthly average patient flow in the hospital is 15,580. The town also has one private primary hospital, three health centres, and nine health posts. Data collection was undertaken between June 8 and December 15, 2022.

### Study population, sample size, and sampling procedure

All diabetes patients aged 18 years and above in the Debre Berhan Comprehensive Specialised Hospital in the diabetes follow-up clinic were the source population. The study population consisted of all newly diagnosed diabetes patients aged 18 years and above from January 1, 2005, to December 31, 2021. Inclusion criteria were all newly diagnosed diabetes patients aged 18 years and above during the study period who had attended follow-up for treatment. Patients who were diagnosed with both diabetes and a foot ulcer simultaneously, as well as patients whose date of DFU was unknown were excluded.

The sample size for this study was calculated using from Open-Epi info software package by considering the desired confidence level = 95%, Power (chance of detecting) = 80%, Ratio of unexposed to exposed as 1 and percentage of outcome among unexposed and exposed to high diastolic blood pressure as 9.67% and 21.7% respectively from the study conducted on Arba Minch Hospital^2^. After adding a 10% buffer for incomplete data, the final sample size was 350. Detailed sample size calculations are available in Supplementary Table S1.

Patients newly diagnosed with type 1 or type 2 DM who had been followed up in Debre Berhan Comprehensive Specialised Hospital were considered in this study and were followed retrospectively. A systematic random sampling technique was used. This approach was chosen due to the large sample frame and to reduce potential clustering by time, while maintaining representativeness. The total number of patients during the recruitment time was 1050. The sampling interval (K) was three, calculated by dividing the total number of patients (*N* = 1050) by the required sample size (*n* = 350). Therefore, K was calculated by the formula, K = N/*n* = 1050/350 = 3. The first sample was selected using the lottery method as a starting point, and it was the second patient. Then every third patient was selected until the predetermined sample size was obtained (in the fashion:−2nd, 5th, 8th …).

### Data collection tools and procedure

The data extraction tool was developed based on the WHO standard tool and adapted by reviewing and modifying various literature sources. The checklist for data extraction consisted of four main components: socio-demographic characteristics, clinical predictors, biological predictors, and time-to-outcome variables. Data for all four components were extracted from patients’ follow-up charts and hospital registers.

The data extraction process was carried out by two BSc nurses under the supervision of a trained supervisor. Patients diagnosed with both diabetes and foot ulcer at the time of initial diagnosis, as well as those whose time of developing diabetic foot ulcer (DFU) was unknown, were excluded from the study.

### Study variables

The outcome variables of this study included incidence (event), loss to follow-up, death, and still on follow-up with no event. These outcome variables were obtained from patient follow-up charts and hospital registers. The outcome variable, DFU, was determined based on physicians’ clinical decisions. Patients who were lost to follow-up, died before the study period ended, or had not developed DFU by the end of the study were considered censored. Therefore, the outcome variables were categorised as either event (DFU) or censored.

The independent variables (predictors) were grouped into three categories. Socio-demographic predictors included age, sex, residence, occupation, marital status, and educational status. Clinical predictors comprised hypertension, body mass index (BMI), type of diabetes mellitus (DM), duration of DM, retinopathy, neuropathy, and nephropathy. These comorbidities were assessed at the time of diabetes diagnosis (baseline) and were based on clinical documentation in the medical records, as determined by physicians’ diagnoses. Biological predictors included high-density lipoprotein (HDL), low-density lipoprotein (LDL), fasting blood sugar (FBS), and triglyceride levels, all measured at the time of diagnosis (baseline). All predictor variables were extracted from patients’ medical charts and hospital records at the time of cohort entry (i.e., the date of diabetes diagnosis), ensuring temporal consistency for the cohort design.

### Operational definitions

**Diabetic foot ulcers** are defined as non-traumatic skin lesions of the skin on the patient`s foot with diabetes mellitus, and their diagnosis will be made after confirmation by a qualified practitioner^2^.

#### Hypertension

systolic and diastolic blood pressure of the person equal to or more than 140 and 90 mmHg, respectively^21^.

#### Newly diagnosed diabetes mellitus

a patient who was diagnosed with diabetes mellitus (type 1 or type 2) for the first time at Debre Berhan Comprehensive Specialised Hospital and enrolled in the diabetes follow-up clinic between January 1, 2005 and December 31, 2021. The follow–up started at the date of first diagnosis.

#### High blood glucose (Hyperglycemia)

fasting plasma glucose concentrations of 126 mg/dl or more, or random plasma glucose or 2-hour post-load glucose concentrations of more than 200 mg/dl^22^.

#### Triglyceride

is normal if its value is < 150 mg/dl and abnormal if its value is ≥ 150 mg/dl.

**LDL (low-density lipoprotein)** is normal if its value is < 100 mg/dl and abnormal if it is ≥ 100 mg/dl.

**HDL (high-density lipoprotein)** is normal if its value is > 40 mg/dl and abnormal if it is ≤ 40 mg/dl^23^.

#### Body mass index (BMI)

weight-for-height is used to classify underweight, overweight and obesity in adults. It is defined as the weight in kilograms divided by the square of the height in meters (kg/m2). BMI < 18.5 kg/m2 = underweight, BMI 18.5–24.9 kg/m2 = normal range, BMI 25–29.9 kg/m2 = overweight and BMI ≥ 30 kg/m2 = obese^24^.

### Data quality assurance

Two trained BSc nurses extracted data using this WHO-based checklist. One BSc nurse supervised the overall data extraction process. The data extractor and supervisor received one day of training on the data extraction techniques. Data extractors ensured that whether or not control had been done on biomedical instruments and whether or not medical apparatuses were calibrated. They also assessed that the results were not a one-off measurement but repeated by another health worker. The data extraction tools were pre-tested on 5% of the calculated sample size two weeks before actual data collection. Ambiguous words and concepts for data extractors were corrected accordingly. Throughout the data extraction, 15% random verification of the extracted data was checked by the supervisor daily for completeness and inter-rater reliability (K = 0.85). The extracted data was reviewed and checked for completeness before data entry.

### Statistical analysis

Data was entered into a computer package through EpiData and then exported to SPSS analysis software for cleaning, coding, categorising, merging and checking for completeness, consistency and outliers. The percentage and frequency of patients regarding all covariates were summarised by descriptive statistics.

The survival experience of the patients was assessed using the Kaplan-Meier survivor function. The independent effect of predictors on the occurrences of diabetic foot ulcers was identified by the Cox proportional hazard model. The potential candidate predictors for the full model were selected by bi-variable Cox proportional hazard regression with a cut-off point of *P* ≤ 0.25.

All variables with a P-value ≤ 0.25 were included in the multivariable analysis. The magnitude of the association was measured by using a hazard ratio with a 95% confidence interval. Statistical significance was declared at P-value ≤ 0.05. Finally, the data was presented with texts, tables and figures.

## Results

### Socio-demographic characteristics of the patients

Data completeness in this study was 91.7%. Of the total 321 study patients, 171 (53.3%) were male. The median age of the patients was 55 years (IQR = 51). Of the 321 patients, 182 (57%) resided in urban areas. Age categories were constructed based on the interquartile distribution of the study sample to ensure balanced subgroup sizes and were further aligned with previous regional studies to facilitate comparability. All socio-demographic characteristics of the study patients can be displayed through the following (Table 1).

**Table 1:** Shows socio-demographic characteristics of adult diabetes patients (*N* = 321).

Variables	Category	Frequency	Percentage
Sex	Male	171	53.3%
	Female	150	46.7%
Age	28–37	15	4.7%
	38–47	68	21.2%
	48–57	103	32.1%
	58–67	84	26.2%
	≥ 68	51	15.9%
Marital status	Single	15	4.7%
	Married	276	86.0%
	Separated	28	8.7%
	Widowed	2	0.6%
Occupation	Private worker	35	10.9%
	Government worker	156	48.6%
	Farmer	113	35.2%
	Housewife	11	3.4%
	Daily labourer	6	1.9%
Educational status	No education	27	8.4%
	Primary education	149	46.45%
	Secondary and above	145	45.25%
Residence	Urban	182	57%%
	Rural	138	43%

### Baseline clinical factors

In this study, the median BMI of the patients was 23.7 kg/m^2^. Among 321 participants, 272 (84.7%) had type 2 DM. Of the total 321 patients, 44 (13.7%) had neuropathy. A total of 198 patients (61.7%) had a normal range of body mass index. The following table displays all the baseline clinical predictors of the patients (Table 2).

**Table 2:** Baseline clinical factors associated with diabetic foot ulcer among the study patients.

Variables	Category	Frequency	Percentage
Type of DM	Type 1	49	15.3%
	Type 2	272	84.7%
Neuropathy	Yes	44	13.7%
	No	277	86.3%
Nephropathy	Yes	4	1.2%
	No	317	98.8%
Hypertension	Yes	222	69.2%
	No	99	30.8
Retinopathy	Yes	41	12.8%
	No	280	87.2%
BMI	Underweight	8	2.5%
	Normal	198	61.7%
	Overweight	86	26.8%
	Obese	29	9%

### Baseline biological factors

Of the total patients of 321, 39 (12.1%) had LDL levels ≥ 100 mg/dl. Regarding triglyceride, 290 (90.3%) had a triglyceride level of < 150 mg/dl. The following table shows the baseline biological predictors of the study patients (Table 3).

**Table 3:** Biological factors of diabetic foot ulcer among patients with diabetes.

Variable	Category	Frequency	Percentage
LDL	Normal (< 100 mg/dl)	282	87.9%
	Abnormal (≥ 100 mg/dl)	39	12.2%
HDL	Normal (> 40 mg/dl)	283	88.2%
	Abnormal (≤ 40 mg/dl)	38	11.8%
Triglyceride level	Normal < 150 mg/dl)	290	90.3%
	Abnormal (≥ 150 mg/dl)	31	9.7%

### Incidence of diabetic foot ulcer

**Fig. 1:**
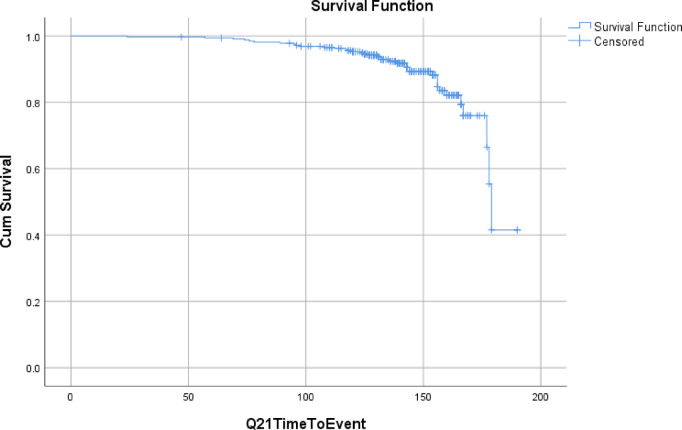
Time-to-event analysis of diabetic foot ulcer incidence.

The incidence rate of diabetic foot ulcer was 1.01 per 100 person-years (95%: CI,.73954_1.39679). Throughout the follow-up period, of 321 study patients, a total of 38 (11.8%) who were free from DFU at the beginning of the follow-up period developed DFU during the follow-up period. Patients were followed for a minimum of 24 months and a maximum of 180 months. The cumulative incidence of DFU was 11.8% over this period. (Fig. 1). Detailed life-table analyses are available in Supplementary Table S2.

### Predictors of diabetic foot ulcer

**Fig. 2:**
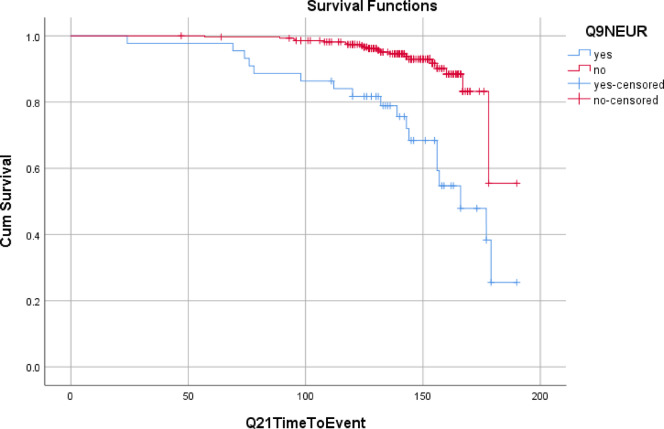
Diabetic foot ulcer-free survival stratified by neuropathy status.

The proportional hazards assumption was checked with the KM curve and Schoenfeld residuals. None of the variables and the global test for the Schoenfeld test was significant (Fig. 2).

### Model fitness

**Fig. 3:**
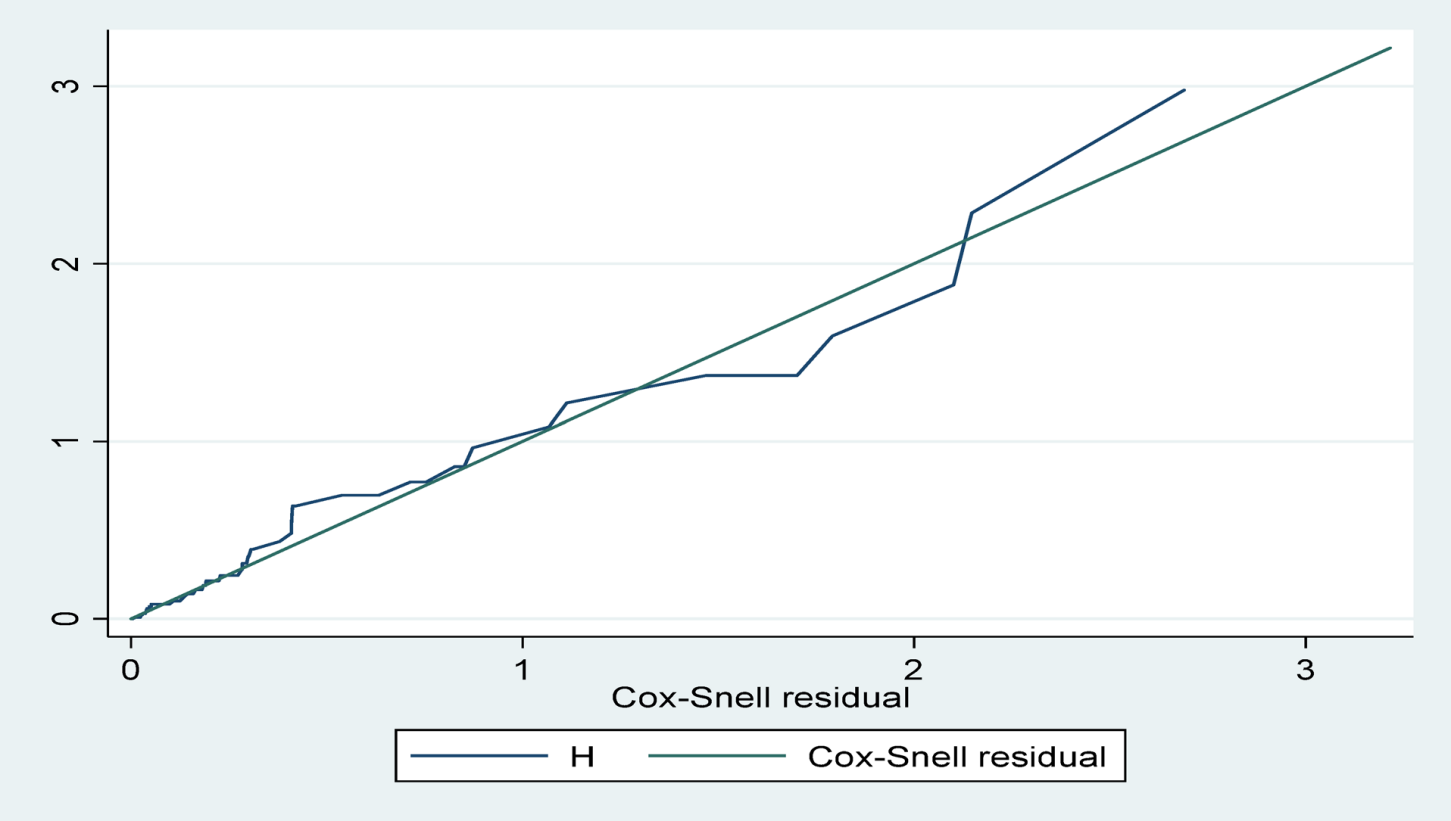
Cox-Snell residuals assessing proportional hazards assumption.

Cox Snell residual plot (Fig. 3).

The cumulative hazard versus Cox-Snell residual plot shows a good model; the residuals with curved lines follow the 45-degree line of the cumulative Hazard (street line).

From the bi-variable Cox regression analysis, variables with a p-value of ≤ 0.25 were selected to be entered into the multi-variable Cox regression analysis. These variables include sex, age category, high-density lipoprotein, Body mass index, neuropathy, residence, hypertension and type of DM. After running the multivariable Cox regression analysis, age, residence, hypertension, neuropathy, body mass index, and high-density lipoprotein remained in the model. However, the sex and category of DM were removed in the variable selection process using the backwards likelihood ratio test.

### Factors associated with diabetic foot ulcer

Following adjustment for potential confounding factors, some factors were significantly associated with the occurrence of DFU. Individuals with abnormal high-density lipoprotein (HDL) levels were 3.778 times more likely to develop DFU compared to those who had normal HDL levels (AHR = 3.713, 95% CI: 1.809–7.620). The presence of neuropathy was 3.411 times more likely to develop DFU compared with neuropathy absence at baseline (AHR = 4.583, 95% CI: 2.367–8.875). Furthermore, participants who were classified as obese, hypertensive, and living in rural areas had a higher risk of developing DFU, with hazard ratios of 2.936 times (AHR = 2.936, 95% CI: 1.107–7.788), 5.609 times (AHR = 5.609, 95% CI: 2.493–12.897) and 2.731 times (AHR = 2.731, 95% CI: 1.268–5.883), respectively, as compared to those who had with normal body weight, non-hypertensive patient, and lived in urban areas. Moreover, patients who were in the age group of ≥ 70 years had a 15.025 times higher hazard of developing DFU as compared with patients of age < 50 years (AHR = 15,025, 95% CI: 3.578–63.038) (Table 4).

**Table 4:** Bi-variable and multivariable Cox regression for predictors of DFU among patients with diabetes.

Variable	Category	Status		CHR (95% CI)	AHR (95% CI)	*P*-Value
		Event	Censored			
Residence	Urban	10	173	1	1	
	Rural	28	110	4.25(2.06–8.76)	2.73(1.27–5.88)	< 0.001
Hypertension	Yes	26	79	4.50 (2.26–8.97)	5.61(2.49–12.90)	< 0.001
	No	12	204	1	1	
Body mass index	Normal	11	193	1	1	
	Overweight	14	73	1.76 (1.11–3.24)	2.13(1.15–3.10)	0.038
	Obese	13	17	2.32(2.15–3.56)	2.94(1.11–7.79)	0.03
Age	< 50	5	115	1	1	
	50–59	8	92	1.71(0.56–5.25)	3.01(0.90-10.07)	0.073
	60–69	20	73	4.04(1.50-10.85)	3.59(1.20-10.72)	0.022
	≥70	5	3	16.29(4.67–56.83)	15.03(3.58–63.04)	< 0.001
Neuropathy	Yes	18	26	4.58(2.37–8.88)	2.94(1.47–5.90)	0.02
	No	20	257	1	1	
HDL level	Normal	19	264	1	1	
	Abnormal	19	19	7.23(3.77–13.88)	3.71(1.81–7.62)	< 0.001

CI: confidence interval; CHR: crude hazard ratio; AHR: adjusted hazard ratio; *p < 0.05; **p < 0.001.

## Discussion

This study revealed that 11.8% (95%, CI 8.4–15.6) of the study patients developed DFU during the follow-up period. This finding is consistent with studies conducted in Australia^25^, the UK^26^, Kenya^27^, and Nigeria^28^. However, the cumulative incidence in this study is higher than that reported in Germany^29^ and Iran^30^. This difference may be attributed to the extended follow-up period in our study. For the study in Germany and Iran, it was three and two years, respectively. As the length of the follow-up period increases, the risk of developing DFU will also increase. Additionally, both the German and Iranian studies were population-based, therefore contributing to the variation in results. Furthermore, the DFU incidence in this study was lower than that of a study conducted in Bahir Dar, Ethiopia^31^ and a systematic review with meta-analysis^32^, The difference may be due to the sample size and length of the follow-up period.

The hazard of diabetic patients developing DFU was 2.7 times higher in rural areas compared to those who live in urban areas. This finding is comparable with the study conducted in Arba Minch^2^, Gondar, Ethiopia^33^, Australia^34^, and the USA^35^. This may be explained by the fact that rural residents may have poor self-care practices and low health-seeking behaviour, and being barefoot while working outdoors can also be another possible explanation^36^.

Individuals in the older age group were 15 times more likely to develop DFU compared to the lower age group patients. This finding is consistent with previous research in Australia^34^, Saudi Arabia^37^, and Portugal^38^. This may be because, as age increases, the rate of wound healing slows because of a decline in the immune system^39^. Furthermore, older patients may have reduced foot self-care practice^40^. Consequently, this increases the risk of DFU.

The hazard of DFU in this study was higher for patients with increased body mass index (BMI) as compared to patients with normal body weight. This finding is in line with the study conducted in Bahir Dar, Ethiopia^31^, Somalia^41^, the UK^42^, and the USA^43^, and a systematic review and meta-analysis^44^. The possible reasons could be that higher BMI was significantly associated with increased prevalence of thin-cap fibroatheroma (TCFA) compared to those with lower BMI. Notably, TCFA is a type of atherosclerotic plaque considered a precursor to plaque rupture and acute coronary thrombosis^45^. Obesity induces chronic inflammation that contributes to atherosclerosis through increased levels of adipokines/cytokines and aldosterone. The adipokines leptin, resistin, IL-6, and monocytes/macrophages recruit immune cells to adipose tissue, triggering systemic inflammation, oxidative stress, insulin resistance, endothelial dysfunction, and pro-thrombotic change, all of which accelerate the atherosclerotic process^46^. This insufficient blood supply lowers wound healing and causes the foot to be at risk of secondary bacterial infections or ischemic ulceration. Additionally, higher pressure on the lower extremities in overweight and obese patients may contribute to the development of foot ulcers^47^.

In this study, patients with neuropathy had a 2.9 times increased risk of developing DFU compared to those without neuropathy. This is consistent with studies conducted in Gondar, Ethiopia^33^, Jimma, Ethiopia^48^, Nigeria^49^, and India^50^. This can be explained by the fact that Diabetes mellitus causes microvascular complications, including neuropathy, which damages the nerve endings in the lower extremities, therefore, affecting an individual’s loss of protective pain sensation, making them unaware of injuries and increasing the risk of foot ulcers^51^. However, this finding was inconsistent with a cross-sectional study conducted in Arba Minch Hospital, which stated that having neuropathy was not significantly associated with the development of DFU^2^. This difference may be due to the difference in length of stay with DM and variations in the study design.

Diabetes patients with hypertension were 5.6 times more likely to develop DFU compared to those without hypertension. This result is similar to other studies done in Somalia^52^ and China^53^, as well as a systematic review and meta-analysis^16^. This might be due to elevated blood pressure, which increases left ventricular (LV) afterload and peripheral vascular resistance. Over time, this sustained pressure results in structural remodelling of the LV due to both pressure and volume overload^54,55^. Heart failure may occur as a result of increased LV stiffness and impaired diastolic function^56^. Hypertension causes heart failure, and then this leads to body swelling, which results in fluid retention in the lower extremities. This prolonged retention of fluid in the lower extremities damages the tissues in that area. Finally, it may lead to ulcers or reduce wound healing^57^.

Our study found that diabetes patients with abnormal high-density lipoprotein (HDL) levels had a 3.7 times higher risk of developing DFU compared to those with normal HDL levels. This finding is supported by studies performed in China and Thailand, as well as a systematic review and meta-analysis^58–60^. Possible reasons could be that HDL has a protective effect against the development of atherosclerosis^61,62^. However, decreased high-density lipoprotein level is related to increased risk of both DM and DFU^63,64^. Lower HDL levels may serve as a potential clinical indicator for predicting lower-extremity amputation and wound-related mortality in patients with DFU^65^. Additionally, some international guidelines recommend different HDL cutoff values for men and women (e.g., < 40 mg/dl for men and < 50 mg/dl for women); our study applied a uniform cutoff of ≤ 40 mg/dl for both sexes. This decision was based on the standard laboratory reference ranges used in the study hospital. We acknowledge that this approach may have underestimated the prevalence of low HDL among female participants and may affect the sex-specific interpretation of this variable.

Finally, in Ethiopia, diabetic foot ulcers represent a growing public health concern^66^. Many patients face barriers such as limited access to wound care services, especially in rural areas where health facilities are under-resourced^67–69^. Additionally, many patients delay seeking care until complications become severe, contributing to high rates of preventable amputations^70^. The pooled prevalence of limb amputation was 31.69%, but the leading contributing factors were trauma, DFU, traditional bone setters, and burns^71^. DFU not only increases the risk of amputation and mortality but also imposes a substantial economic burden on families and the healthcare system through prolonged hospital stays, loss of productivity, and long-term disability^72–75^.

### Strengths and limitations of the study

Our retrospective cohort study established a temporal relationship between the outcome variable and independent variables, and the findings reported are useful for both patients and health professionals. The study identifies and discusses significant predictors with a strong association with DFU occurrence to guide targeted interventions to enhance prevention strategies and reduce complications such as financial, physical, and psychological consequences. Nonetheless, the study was limited to some extent. It did not quantify factors such as physical activity, foot care habits, smoking, and alcohol consumption, which are relevant to the development of DFUs. It also relied on clinical and biological measurements at baseline, which are time-dependent and may change over time. Since the study used secondary data, it was difficult to obtain all the information we needed. Although consecutive sampling is often preferred for retrospective cohort designs, we used systematic random sampling to manage the large eligible sample and reduce selection bias. Additionally, our variable selection was guided primarily by statistical significance rather than biological or clinical plausibility. While this approach ensured objectivity in variable entry, it may have overlooked important predictors supported by clinical evidence. Future prospective studies should incorporate more clinically guided model-building strategies.

## Conclusions

The research indicated that the occurrence of diabetic foot ulcers (DFUs) was considerably lower throughout the follow-up period, which may be attributed to hospital-based health education efforts that promoted good glycemic control and improved medication adherence among patients. Nonetheless, some of the significant risk factors, including obesity, hypertension, old age, rural residence, abnormal HDL levels, and neuropathy, remarkably increased the risk of developing DFU. To mitigate its impact, high-risk patients require close monitoring, early screening, and early therapeutic management. Regular screening and focused preventive measures should be given the most attention by health professionals, and hospitals need to make sure that qualified personnel and necessary medical supplies are available. Education should also be provided to patients regarding DFU risk factors, potential complications, and the importance of timely medical attention. Physicians must prioritise screening and control of the key DFU predictors, whereas health workers need to reinforce patient education. The Ministry of Health and non-government organisations are to incorporate diabetes and its complications into the national health policy, with DFU as a public health issue. Healthcare systems strengthening and risk reduction measure enhancement will be crucial in the prevention of DFU and its serious complications, such as amputations. In addition, behavioural determinants like drinking alcohol, smoking, and physical inactivity require further investigation to help improve DFU prevention and management.

## Supplementary Information

Below is the link to the electronic supplementary material.

Supplementary Material 1

## Data Availability

Data are available upon reasonable request. The full data set used in this study is available from the first author at yohannesterefe3@gmail.com. However, reanalysis of the full data for other use requires approval by Debre Berhan University, Asrat Weldeyes Health Science Campus.

## Abbreviations

WHO: World health organization
BMI: Body mass index
BSC: Bachelor of science
CI: Confidence interval
DFU: Diabetic foot ulcer
DM: Diabetes mellitus
EB: Ethiopian birr
HDL: High-density lipoprotein
KG/M^2^: Kilo gram per meter squared
LDL: Low-density lipoprotein
MG/DL: Milligram per deciliter
MMHG: Millimeter of mercury
SPSS: Statistical package for social sciences
SBP: Systolic blood pressure
BP: Blood pressure
FBS: Fasting blood sugar
DBU: Debre Berhan University
