# Supplementary material for: Incidence of diabetic foot ulcer and its predictors among adult diabetes patients in Northern Ethiopia: a retrospective cohort study
Source: Sci Rep. 2025 Oct 10;15:35466. doi: 10.1038/s41598-025-19253-7 (PMC12514279; doi:10.1038/s41598-025-19253-7)
Supplement: Supplementary file 1 — Supplementary Material 1 [file 41598_2025_19253_MOESM1_ESM.docx]

Supplementary Material

# Supplementary Tables

Table 1: Sample size calculation for predictor analysis using Open-Epi software.

| Factors | % of outcomes among exposed | % of outcomes among unexposed | Reference | Calculated sample size |
| --- | --- | --- | --- | --- |
| Comorbidity | 24.34% | 4% | A study conducted in (2) | 110 |
| Occupation | 29.16% | 4.4% | A study conducted in (2) | 86 |
| Diastolic BP | 21.7% | 9.67 | A study conducted in (2) | 318 |
| The largest calculated sample (318 for diastolic BP) was selected for robustness. With a 10% non-response rate, the final sample was 350. | | | | |

Table 2: Life-table analysis of diabetic foot ulcer incidence by 12-month intervals.

| Interval | Beg. Total | Deaths | Lost | Survival | Std.  Error | [95% Conf. Int.] | |
| --- | --- | --- | --- | --- | --- | --- | --- |
| 24-36 | 321 | 1 | 0 | 0.9969 | 0.0031 | 0.9781 | 0.9996 |
| 36-48 | 320 | 0 | 1 | 0.9969 | 0.0031 | 0.9781 | 0.9996 |
| 48-60 | 319 | 1 | 0 | 0.9938 | 0.0044 | 0.9753 | 0.9984 |
| 60-72 | 318 | 1 | 1 | 0.9906 | 0.0054 | 0.9712 | 0.9970 |
| 72-84 | 316 | 3 | 0 | 0.9812 | 0.0076 | 0.9587 | 0.9915 |
| 84-96 | 313 | 2 | 1 | 0.9749 | 0.0087 | 0.9505 | 0.9874 |
| 96 – 108 | 310 | 2 | 6 | 0.9686 | 0.0098 | 0.9424 | 0.9830 |
| 108-120 | 302 | 4 | 11 | 0.9555 | 0.0116 | 0.9260 | 0.9734 |
| 120-132 | 287 | 5 | 76 | 0.9363 | 0.0142 | 0.9018 | 0.9590 |
| 132-144 | 206 | 6 | 62 | 0.9042 | 0.0188 | 0.8599 | 0.9351 |
| 144-156 | 138 | 3 | 60 | 0.8791 | 0.0232 | 0.8249 | 0.9174 |
| 156-168 | 75 | 7 | 51 | 0.7548 | 0.0479 | 0.6455 | 0.8346 |
| 168-180 | 17 | 3 | 12 | 0.5489 | 0.1072 | 0.3214 | 0.7284 |
| 180-192 | 2 | 0 | 2 | 0.5489 | 0.1072 | 0.3214 | 0.7284 |
